# The Effect of Glass Fiber Powder on the Properties of Waterborne Coatings with Thermochromic Ink on a Chinese Fir Surface

**DOI:** 10.3390/polym11111733

**Published:** 2019-10-23

**Authors:** Xiaoxing Yan, Xingyu Qian, Yijuan Chang, Rong Lu, Tetsuo Miyakoshi

**Affiliations:** 1 College of Furnishings and Industrial Design, Nanjing Forestry University, Nanjing 210037, China; yanxiaoxing@njfu.edu.cn (X.Y.); qianxingyu@njfu.edu.cn (X.Q.); changyijuan1995@163.com (Y.C.); 2 Department of Applied Chemistry, School of Science and Technology, Meiji University, 1-1-1 Higashimita, Tama-ku, Kawasaki-shi 214-8571, Japan; miya@meiji.ac.jp

**Keywords:** glass fiber powder, thermochromic ink, waterborne coating, film performance

## Abstract

In this study, the effect of glass fiber powder on the properties of waterborne coatings with thermochromic ink was investigated, using Chinese fir board as the base material and temperature-sensitive thermochromic waterborne coatings with thermochromic ink as the paint base. The concentration of glass fiber powder was determined when the microstructure, optical properties, mechanical properties, liquid resistance, and heat preservation effect were the best. The results showed that the paint film with glass fiber powder concentration of 1.0% to 7.0% had better discoloration performance. With an increase of the glass fiber powder concentration, the gloss of the paint film decreased gradually, and when the concentration of glass fiber powder was 0% to 5.0%, the gloss of the paint film was better. The concentration of glass fiber powder had no effect on the adhesion, impact resistance, and liquid resistance. In the first 2.5 min, the temperature value of the waterborne coating with 3.0% glass fiber powder was higher than that without glass fiber powder, which has a certain heat preservation effect. When the glass fiber powder content was 3.0%, the microstructure of paint film was the best. The composition of paint film with different glass fiber powder concentrations was not different and the discoloration performance of paint film with heat preservation effect was not affected by time. The analysis showed that the waterborne coating with 3.0% glass fiber powder had the best comprehensive performance. This work provides a technical reference for the industrialization of heat preservation and thermochromic coating on wood.

## 1. Introduction

As a special coating which can change the color of many materials [1], thermochromic coatings have attracted wide attention of scientists in the fields of surface temperature measurement, chemical anti-counterfeiting, entertainment decoration, and other fields [2,3]. There are many ways to prepare thermochromic coatings, which can be classified as inorganic, organic, and liquid crystal [4]. These thermochromic materials are mainly used in paper and printing materials, but little research has been done on thermochromic materials for the coatings on the surface of wood. Thermochromic coatings on wood surface can change the color with the change of temperature, make the products show different colors with the change of season, and make the products more humanized and changeable. It can not only change the color, but also can give people different feelings. The method of preparing thermochromic coatings by adding thermochromic ink is convenient, fast, and beautiful, and it can make the product surface have better effect. Therefore, it is widely used in printing and spraying fields [5,6]. In addition, among many kinds of coatings, waterborne coatings have attracted more and more attention in the market due to their advantages of no toxicity, environmental protection, good adhesion, and good brushing effect for interior coatings [7,8], however, temperature-sensitive thermochromic coatings are obviously affected by temperature [9], and waterborne coatings have low hardness, poor wear resistance, and other defects [10].

Glass fiber powder is a kind of inorganic non-metallic material, which is long rod in microstate and consists of many kinds of oxides [11]. Because of its high mechanical strength, excellent heat resistance, good insulation, low moisture absorption, and corrosion resistance [12], it has been widely used in insulation materials, plastics, rubber, cement, and other fields [13], and is often used as automotive wall materials due to its thermal insulation performance [14]. Gao et al. [15] used glass fiber and Al powder to modify glass-ceramics. The effects of varying quantities of glass fiber and Al powder on the microstructures and properties of glass-ceramics were studied. The experimental results showed that glass fiber can significantly improve the mechanical strength of glass-ceramics. Cavasin et al. [16] prepared a kind of epoxy foam for thermal insulation of glass fiber reinforced plastics by studying different formulations and curing parameters and its mechanical properties, thermal conductivity and microstructure were tested. The results showed that there was a continuous interface between the epoxy glass fiber reinforced plastics and the foaming layer, and the foaming also affected the mechanical properties and thermal conductivity of the material. The research on glass fiber powder mainly focused on the modification of composite materials, and there were few reports about its application in the field of coatings on the surface of wood. In this study, a waterborne thermochromic coating was prepared by adding water-based paint with thermochromic ink, and glass fiber powder was added to explore the effect of different concentrations of glass fiber powder on its properties, and whether the waterborne thermochromic coatings with glass fiber powder had heat preservation effect. The effect of time on the discoloration performance of paint film with heat preservation effect was also investigated to establish a foundation for the application of thermochromic coatings on interior wood products.

## 2. Experiment Materials and Methods

### 2.1. Experimental Materials

The main components of thermochromic ink are methyl red (as leuco agent, MW: 269.30 g/mol, CAS No. 493-52-7), bisphenol A (as chromogenic reagent, MW: 228.29 g/mol, CAS No. 80-05-7), and melamine (MW: 126.12 g/mol, CAS No. 108-78-1), which were supplied by Huancai Discoloration Technology Co., Ltd., Shenzhen, China. Tetradecanol (as co-solvent, MW: 214.39 g/mol, CAS No. 112-72-1), 37.0% formaldehyde solution (MW: 30.03 g/mol, CAS No. 50-00-0), sodium dodecyl benzene sulfonate (MW: 348.48 g/mol, CAS No. 25155-30-0), triethanolamine (MW: 149.19 g/mol, CAS No. 102-71-6), citric acid monohydrate (MW: 210.14 g/mol, CAS No. 5949-29-1) and anhydrous ethanol (MW: 46.07 g/mol, CAS No. 64-17-5) were supplied by Xilong Chemical Co., Ltd., Guangzhou, China. A waterborne acrylic copolymer dispersion was supplied by Yihua Lifestyle Technology Co., Ltd., Shantou, China. The waterborne coatings were composed of waterborne acrylic copolymer dispersions (concentration of 90.0%), matting agents (concentration of 2.0%), additives (concentration of 2.0%), and water (concentration of 6.0%), with a solid concentration more than 30.0%. Chinese fir boards (100 mm × 100 mm × 12 mm, uniform material color, 300 pieces, after ordinary mechanical sanding) were supplied by Yihua Lifestyle Technology Co., Ltd., Shantou, China. The studied wood samples had a radial sawn wood texture. Glass fiber powder (800 meshes in fineness, mainly composed of SiO_2_, CaO, Al_2_O_3_, FeO) were supplied by Guangzhou Fuhua New Material Co., Ltd., Guangzhou, China. The NaCl solution (concentration of 15.0%) and medical ethanol (concentration of 70.0%) were supplied by Otopp Biotechnology Co., Ltd., Hangzhou, China. Detergent was supplied by Shanghai Hutchison Whitecat Co., Ltd., Shanghai, China. Red ink was supplied by Fine Stationery Co., Ltd., Shanghai, China.

### 2.2. Preparation of Ink

#### 2.2.1. Preparation of the Wall Material Prepolymer

The thermochromic microcapsules were prepared by in situ polymerization. The 5.0 g melamine, 10.0 g 37.0% formaldehyde solution, and 10.0 mL deionized water were poured into a 250 mL beaker. Triethanolamine was slowly added to adjust the pH value of the solution to the range, 8.5–9.0. Then, the mixture was put into a 70 °C constant-temperature water bath with stirring at 700 rpm to obtain a transparent wall material solution.

#### 2.2.2. Preparation of Thermochromic Emulsion for Core Material

One gram of sodium dodecylbenzene sulfonate was dissolved completely in 99.0 g deionized water to obtain 1.0% emulsifier solution. Then 1.0 g methyl red, 1.0 g bisphenol A, and 10.0 g tetradecanol were added to the 1.0% emulsifier solution, and the beaker was put into a water bath pan. The temperature was 60 °C and the speed was 1200 rpm, stirring for 30 min continuously to obtain stable core material emulsion.

#### 2.2.3. Preparation of Thermochromic Ink

Then, the cooled wall material solution was added into the core material emulsion and stirred evenly. The citric acid monohydrate was added to adjust the solution pH to between 4.0 and 5.0. Reaction was carried out at 60 °C for 3 h in a water bath and then cooled to room temperature. The product was filtered and washed repeatedly with deionized water and absolute ethanol. Then, it was dried in an oven at 25 °C for 24 h, and 6.5 g of thermochromic microcapsules were obtained. The thermochromic microcapsules were added to 12.0 g waterborne acrylic copolymer dispersion, and they were mixed evenly to obtain the thermochromic ink.

### 2.3. Preparation of Coatings

First, Chinese fir boards were placed at room temperature and a relative humidity of 50.0% ± 5.0% for one week to balance the moisture concentration of the substrate. One gram of glass fiber powder and 15.0 g thermochromic ink were added to 84.0 g waterborne topcoat, mixed evenly, and the waterborne topcoat with the mass fraction of 1.0% glass fiber powder was prepared. The ingredients of the waterborne coatings with thermochromic ink and glass fiber powder are listed in Table 1. Other proportions of 3.0%, 5.0%, 7.0%, and 10.0% were also produced according to this method. The waterborne primer was coated on the base material using an SZQ tetrahedral fabricator (Tianjin Jinghai Science and Technology Testing Machinery Factory, Tianjin, China) and dried at room temperature for 30 min, and then the sample was transferred to a 35 °C oven. After the weight of the samples remained unchanged, it was taken out and naturally cooled to room temperature. The coatings were sanded with 800 grit sandpaper, and the dust was wiped off with a dry cloth. The coating process of the primer was repeated three times. The layer number of the primer was 3 and the layer number of the topcoat was 3. The finishing method of topcoat was the same as the above, and it was also coated three times. The thickness of the dry waterborne coating film was about 60 μm.

### 2.4. Testing and Characterization

The microstructures of thermochromic ink, glass fiber powder, and waterborne coatings were observed using a Quanta 200 environment scanning electron microscope (SEM) (FEI Company, Hillsboro, OR, USA). The components of glass fiber powder and waterborne coatings with thermochromic ink and different concentrations of glass fiber powder were analyzed using a Vertex 80V infrared spectrum analyzer (Germany Bruker Co., Ltd., Karlsruhe, Germany). According to the standard of GB/T 9754-2007 [17], the gloss of waterborne coating films at 20°, 60° and 80° was measured by an HG268 gloss meter (3NH Technology Co., Ltd., Shenzhen, China). The CIELAB color values of the waterborne coating films was measured by a HP-2136 colorimeter (Zhuhai Tianchuang Instrument Co., Ltd., Zhuhai, China). The change of chromatic values of waterborne coatings with thermochromic ink and different concentrations of glass fiber powder from 18 to 40 °C was tested by the colorimeter. *L*, *a*, *b*, *C*, and h are expressed as lightness, change of red and green color, change of yellow and blue color, color chroma, and hue, respectively. Δ*L* (brightness difference) = *L*_1_ − *L*_2_, Δ*a* (red and green difference) = *a*_1_ − *a*_2_, and Δ*b* (yellow blue difference) = *b*_1_ − *b*_2_. *L*_1_, *a*_1_, and *b*_1_ are all initial values (18 °C), whereas *L*_2_, *a*_2_, and *b*_2_ are the values after the temperature change. The color difference was calculated by the following Formula (1) [18]:

Δ*E* = [(Δ*L*)^2^ + (Δ*a*)^2^ + (Δ*b*)^2^]^1/2^ (1)

The waterborne coatings with thermochromic ink and different concentrations of glass fiber powder were heated on an HHP1 heating plate (Shanghai Hengyue Medical Devices Co., Ltd., Shanghai, China). While the coating was slowly heated, the surface temperature of the coating was measured by a temperature sensor, and at the same time the color values of the coating were measured by the colorimeter to determine the temperature dependence of the results. According to GB/T 1720-89 [19], a QFZ-II coating adhesive tester (Tianjin JingKelian Material Testing Machine Co., Ltd., Tianjin, China) was used to determine the adhesion of the coating. After the test, the damage of the coating was observed with a quadruple magnifying glass. According to the damage situation of the coating, it could be divided into 7 levels. Level 1 indicated the best adhesion, and level 7 indicated the worst adhesion. According to the standard of GB/T1732-93 [20], the impact strength was measured by a QCJ impactor (Tianjin Jingkelian Material Testing Machine Co., Ltd., Tianjin, China). During the testing process, the sample coating was put on the anvil at the bottom of the instrument, a 1.0 kg ball was raised to a certain height (the maximum height was 50 cm), then, the ball was freely dropped to impact the sample, and observed with a magnifying glass to determine whether the coating had cracks, wrinkles, and peeling. The hardness of the coating was measured by a coating hardness tester with 6H-6B pencil (Kunshan Jingjia Instrument Equipment Co., Ltd., Kunshan, China). In the hardness test, the angle between the pencil and the coating was 45°, and the pencil scratched under a 1.0 kg load. The hardness of the coating (determined with 6H, 5H, 4H, 3H, 2H, 1H, HB, 1B, 2B, 3B, 4B, 5B, and 6B pencils) was measured when scratches appeared on the coatings. The hardness of the pencil represented the hardness of the coating. According to GB/T 1733-93 [21], the liquid resistance of the coating was measured using 15.0% NaCl solution, 70.0% medical ethanol, detergent, and red ink. After the filter paper was soaked in the test solutions, it was taken out with a tweezer and placed in the test area of the coating. The sample was covered with a glass cover. After 24 h, the glass cover and filter paper were removed. The residual liquid on the coating surface was absorbed, and the imprint and discoloration were checked after a period of time. The aging and stability test were measured in the ZN ultraviolet weather resistance tester (Nanjing Environmental Test Equipment Co., Ltd., Nanjing, China). A mixture of rosin and paraffin was coated on the side and the back of the Chinese fir boards, leaving a 10.0 × 5.0 cm^2^ waterborne wood coating, and the samples were put in the ZN ultraviolet weather resistance tester for 200 h. All the experiments were repeated four times with an error of less than 5.0%.

## 3. Results and Discussion

### 3.1. Microstructure Analysis

**Figure 1:**
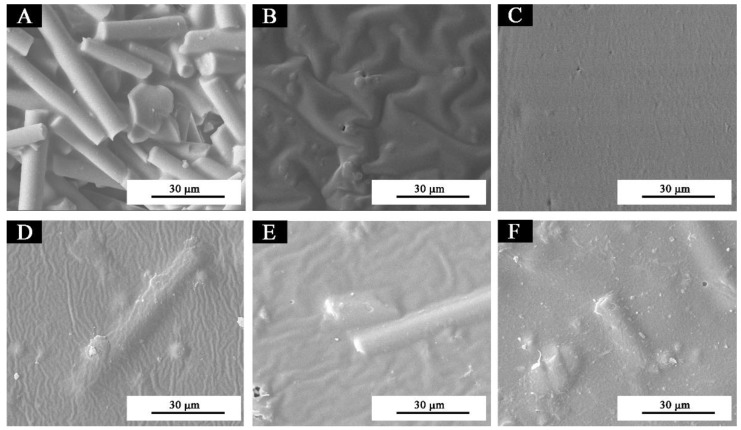
SEM of the waterborne coatings with thermochromic ink and different concentrations of glass fiber powder: (**A**) glass fiber powder, (**B**) thermochromic ink, (**C**) pure coating, and (**D**–**F**) 1.0%, 3.0%, and 10.0% glass fiber powder in the waterborne coating with thermochromic ink, respectively.

**Figure 2:**
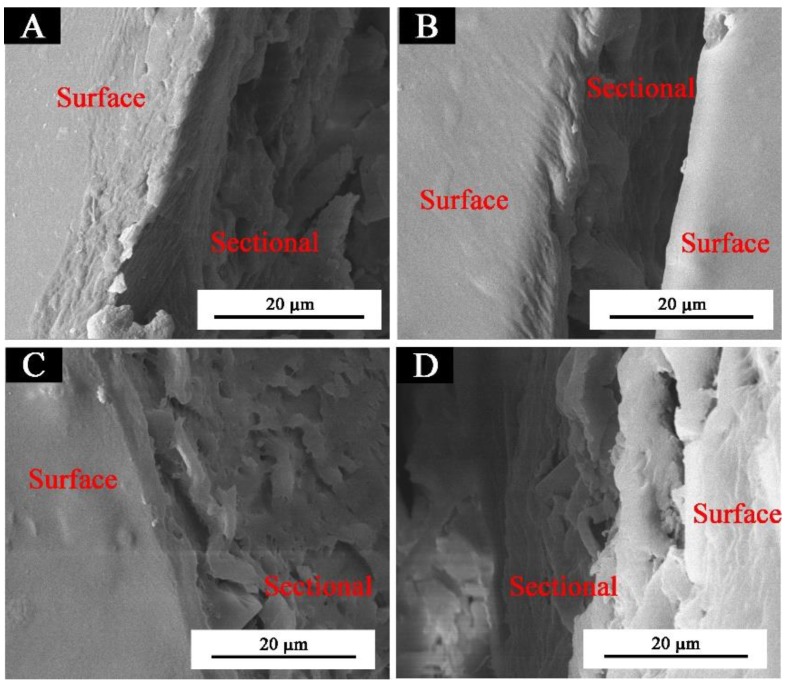
Cross-sectional SEM of the waterborne coatings with thermochromic ink and different concentrations of glass fiber powder: (**A**) pure coating and (**B**–**D**) 1.0%, 3.0%, and 10.0% glass fiber powder in the waterborne coating with thermochromic ink, respectively.

**Figure 3:**
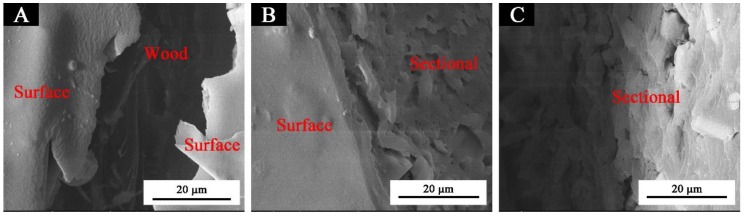
Cross-sectional SEM of the waterborne coating with thermochromic ink and 3.0% of glass fiber powder: (**A**) coating surface and wood, (**B**) surface and section, and (**C**) sectional micrographs.

Figure 1 shows that the glass fiber powder was a columnar structure in the microstructure (Figure 1A), and the pure coating without thermochromic ink or glass fiber powder was smooth and almost free of particles (Figure 1C). There were obvious particles in the waterborne coating with 1.0%, and 3.0% glass fiber powder, which were uniformly distributed without agglomeration, however, with an increase of the concentration of glass fiber powder, the columnar glass fiber powder increased significantly. When the concentration was 10.0%, the fibers were more and agglomerate. The main reason was that an increase of glass fiber powder concentration led to an increase of agglomeration of columnar fibers, which was also the reason for a decrease of gloss of paint film [22] and an increase of hardness of paint film [23]. The cross-sectional SEM micrographs of the pure coating and the coatings with different concentrations of glass fiber powder are shown in Figure 2 and Figure 3. Observations of the cross-sectional SEM show that 3.0% fiber glass powder was evenly distributed in the coating and not impregnated in the wood structure. The results showed that the coating film had the best microstructure when the glass fiber powder concentration was 3.0%.

### 3.2. Effect of Glass FiberPpowder Concentration on OpticalPproperties

**Figure 4:**
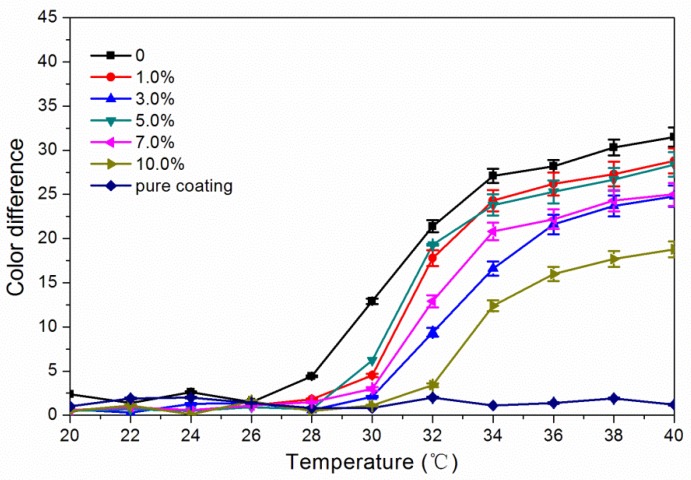
Effect of glass fiber powder concentration on the color difference of waterborne coatings with thermochromic ink from 18 to 40 °C.

The results of chromatic values of waterborne coatings with thermochromic ink and different concentrations of glass fiber powder from 18 to 40 °C are shown in Table 2. The results of color difference are shown in Figure 4. When the temperature rose from 18 to 28 °C, the color difference of the waterborne coatings with thermochromic ink and different concentrations of glass fiber powder was 0.1 to 4.4, and there was no obvious discoloration effect. When the temperature continued to rise to 30 °C, the paint film without glass fiber powder began to discolor, and the color difference was 12.9. The coating with 1.0% to 7.0% glass fiber powder began to change color obviously when it was heated to 32 °C, and the color of the paint film with glass fiber powder concentration of 10.0% did not change significantly until 34 °C. The overall trend of Figure 4 shows that at the same temperature, the color difference of waterborne coatings without glass fiber powder was the greatest, and that the color difference of waterborne coatings with glass fiber powder concentration of 10.0% was the smallest. Glass fiber powder was white, which would affect the discoloration effect, thus affecting the color difference. In addition, when the concentration of glass fiber powder was high, it was easy to agglomerate in the coating, not easy to disperse, and the color difference was also affected [24]. Therefore, in the heating process, the color difference of the waterborne coating with glass fiber powder was less than that without glass fiber powder, and discoloration needs higher temperature. When the temperature was 34 °C, the color difference of the coatings with different concentrations of glass fiber powder was high. The waterborne coatings with thermochromic ink and a glass fiber powder concentration of 1.0% to 7.0% had better discoloration performance.

In this experiment, three incident angles were used to reflect the effect of glass fiber powder concentration on the gloss of the waterborne coatings with thermochromic ink, which were 20°, 60°and 85°, respectively. As shown in Table 3, the greater the incident angle, the higher the gloss. Under the same incident angle, the gloss of waterborne coatings decreased with an increase of glass fiber powder concentration. This may be due to an increase of glass fiber powder concentration, which increased the roughness of the paint film surface and produced diffuse reflection on the paint film surface. When the concentration of glass fiber powder was 0% to 5.0%, the gloss of the waterborne coatings with thermochromic ink was good.

### 3.3. Effect of Glass Fiber Powder Concentration on Mechanical Properties

The adhesion of the coating represents the bonding strength between the coating and the substrate, and the coating with good adhesion can play a protective and decorative role [25]. Impact resistance represents the ability of the film to resist impact loading [26]. The hardness of the coating is the ability of the coating to resist the external mechanical effects such as collision, scratching, and so on [27]. The effects of glass fiber concentration on the adhesion, impact resistance, and hardness of waterborne coatings with thermochromic ink are shown Table 4 and Table 5. The adhesion level of waterborne coatings with different concentrations of glass fiber powder was 0, and the impact resistance of waterborne coatings with different concentrations was 5.0 kg·cm, which was higher than that of pure coating without thermochromic ink. The adhesion and impact resistance did not change with the concentration of glass fiber powder, so the concentration of glass fiber powder had no effect on the adhesion and impact resistance of the paint film. The hardness of the pure coating was HB, and with an increase of glass fiber powder concentration, the hardness of the coating increased gradually indicating that the thermochromic ink and glass fiber powder have certain effect on the hardness of the coating [23].

### 3.4. Effect of Glass Fiber Powder Concentration on Liquid Resistance

The waterborne coatings with thermochromic ink and different concentrations of glass fiber powder were tested using four kinds of solution including NaCl solution, medical ethanol, detergent, and red ink at 18 °C. The chromatic value of the paint film without liquid resistance test was recorded, and the chromatic value, color difference, and liquid resistance level of the paint film after 24 h for testing using four kinds of test solution were also recorded. The results are shown in Table 6. The results of color difference and liquid resistance level are shown in Table 7 and Table 8. It was shown that the liquid resistance level of the paint film with a glass fiber powder concentration of 0% to 10.0% on NaCl solution, medical ethanol, and detergent was level 1 and without impression. The red ink resistance level of the pure coating was level 1 and that of the paint film with glass fiber powder concentration of 0% to 10.0% was level 3, with obvious marks. The color difference of the coatings with glass fiber powder was more obvious after the liquid resistance test of red ink, which proved that the red ink resistance of the coatings with glass fiber powder was worse. The reason may be that the permeability of red ink increased after adding glass fiber powder [28]. The liquid resistance level of the paint film did not change with a difference in the glass fiber powder concentration, which proved that the glass fiber powder concentration had no effect on the liquid resistance level of waterborne coatings with thermochromic ink.

### 3.5. Effect of Glass Fiber Powder Concentration on Temperature Change of Waterborne Coatings

**Figure 5:**
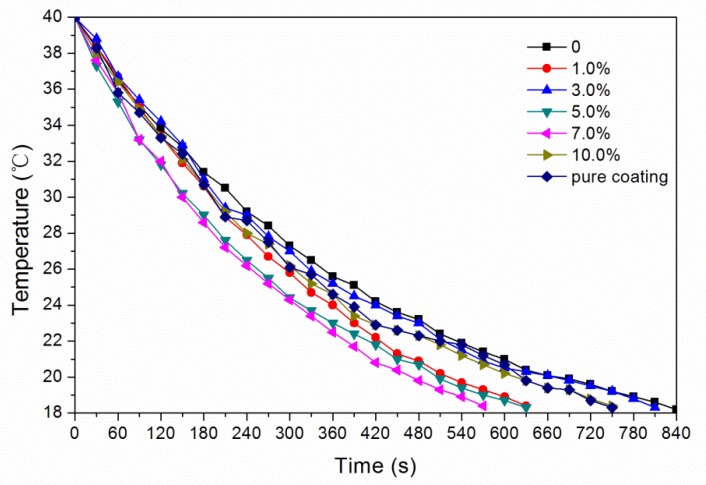
Effect of glass fiber powder concentration on temperature change of naturally cooled waterborne coatings with thermochromic ink.

In order to test the temperature change of waterborne coatings with thermochromic ink and different concentrations of glass fiber powder, the samples of film with different concentrations of glass fiber powder were heated to 40 °C using a heating plate, and then cooled to 18 °C. During this period, the temperature of the film was measured by the temperature sensor every 30 s and stopped at 18 °C. The variation of the temperature of films with different concentrations of glass fiber powder is shown in Figure 5. Figure 5 shows that the waterborne coatings with 1.0%, 5.0%, 7.0%, and 10.0% glass fiber powder had lower temperature value and faster heat dissipation than that without glass fiber powder. The temperature of the waterborne coating with 3.0% glass fiber powder was higher than that without glass fiber powder in the first 2.5 minutes, which had a certain heat preservation effect for interior wood coatings. The reason may be that in the waterborne coating with thermochromic ink, the ink was liquid and could be evenly coated on the surface of Chinese fir board. When glass fiber powder was added, the heat preserved in glass fiber powder was less than that lost from the gap between glass fiber powder.

### 3.6. Infrared Spectrum Analysis

**Figure 6:**
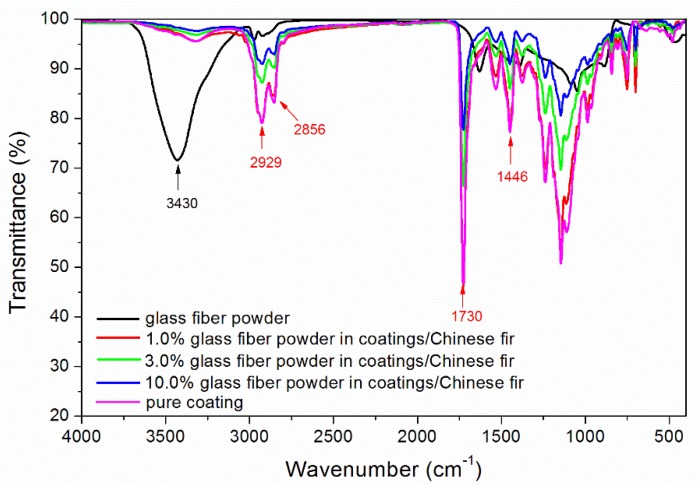
Infrared spectra of different concentrations of glass fiber powder in the waterborne coatings with thermochromic ink.

Infrared spectroscopy tests were carried out on glass fiber powder, pure coating, and different concentrations of glass fiber powder in the waterborne coatings with thermochromic ink on Chinese fir (Figure 6). As can be seen from Figure 6, 3430 cm^−1^ was –OH absorption peaks. When the concentration of glass fiber powder increased, the absorption of hydroxyl group decreased, which indicated that the dehydration reaction took place between glass fiber powder and waterborne coatings with thermochromic ink [29]. It may be that CaO in glass fiber powder reacted with hydrogen peroxide to absorb water [30]. The 2929, 2856, and 1446 cm^−1^ were –CH_2_ absorption peaks; and 1730 cm^−1^ was a strong and sharp characteristic peak of the C=O group. Infrared spectroscopy showed that there were no peaks disappearing or appearing with a change of glass fiber powder concentration, which indicated that there was no difference in the composition of the waterborne coatings with different glass fiber powder concentrations.

### 3.7. Stability of Thermochromic of Coatings

In order to test the effect of time on the color values of the waterborne coating with thermochromic ink and 3.0% glass fiber powder with a certain heat preservation, samples of waterborne paint film with 3.0% glass fiber powder were placed for three months, and their chromatic values were tested. As shown in Table 9, the chromatic values of the film after three months were measured at 18 °C and three consecutive days in a 30 °C oven. Table 10 shows that the color difference of the film with 3.0% glass fiber powder was 1.3 to 1.6 at 18 °C and 30 °C for three consecutive days after three months, and there was no obvious color change. This showed that the time had no effect on the chromatic value of the waterborne coating with heat preservation effect after adding 3.0% glass fiber powder and the discoloration effect of waterborne wood coating was stable.

**Figure 7:**
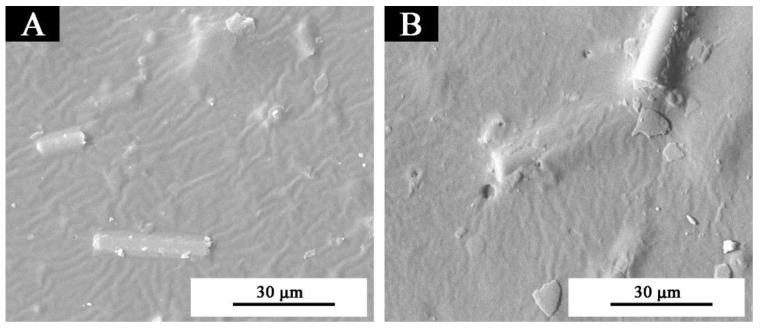
SEM of waterborne wood coatings with 3.0% glass fiber powder: (**A**) before and (**B**) after aging.

**Figure 8:**
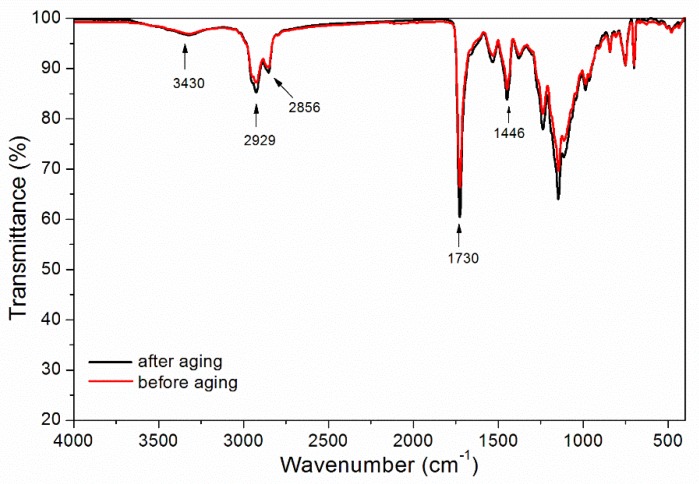
Infrared spectra of waterborne wood coatings with 3.0% glass fiber powder before and after aging.

The waterborne wood coatings with thermochromic ink and 3.0% glass fiber powder underwent the ultraviolet weather resistance test for 200 h. The chromatic values and gloss of the coating after aging were measured (Table 11). The waterborne wood coatings were analyzed by SEM (Figure 7) and infrared spectroscopy (Figure 8). The results showed that after ultraviolet-accelerated aging, the color difference of the waterborne wood coatings was 3.4, which is slight discoloration. The gloss of the waterborne wood coatings containing 3.0% glass fiber powder were basically unchanged. The infrared characteristic peaks remained unchanged, and no cracking was observed on SEM, which indicated that the waterborne wood coatings had good stability and aging resistance during the ultraviolet weather resistance test.

## 4. Conclusions

In this study, the performances of waterborne coatings with thermochromic ink and different concentrations of glass fiber powder were tested, and the effects of glass fiber powder on the properties of waterborne coatings with thermochromic ink were explored. The experimental results showed that the color difference of waterborne coatings with thermochromic ink was less than that without adding glass fiber powder, and the discoloration needed higher temperature. The waterborne coatings with 1.0% to 7.0% glass fiber powder had better discoloration ability. Under the same intensity of incident light, the gloss of the coating decreased gradually with an increase of glass fiber powder concentration, and when the concentration of glass fiber powder was 0% to 5.0%, the gloss of paint film was better. The concentration of glass fiber powder had no effect on the mechanical properties and liquid resistance of the paint film. The temperature value of the waterborne coating with glass fiber powder was higher than that without glass fiber powder in the first 2.5 minutes, which had a certain heat preservation effect. When the concentration of glass fiber powder content was 3.0%, the coating had the best microstructure, and there was no difference in the composition of waterborne coatings with different glass fiber powder concentrations. The color difference of the waterborne coating with heat preservation effect was not affected by time. This study showed that the waterborne coating with 3.0% glass fiber powder had the best comprehensive performance. This study laid a foundation for intelligent thermochromic and heat preservation coatings on an interior wood surface.

## Figures and Tables

**Table 1:** Ingredients of the waterborne coating with thermochromic ink and glass fiber powder.

Concentration of Glass Fiber Powder (%)	Weight of Glass Fiber Powder (g)	Weight of Thermochromic Ink (g)	Weight of Waterborne Primer (g)	Weight of Waterborne Topcoat (g)	Weight of Waterborne Thermochromic Coating (g)
0	0	15.0	100.0	85.0	200.0
1.0	1.0	15.0	100.0	84.0	200.0
3.0	3.0	15.0	100.0	82.0	200.0
5.0	5.0	15.0	100.0	80.0	200.0
7.0	7.0	15.0	100.0	78.0	200.0
10.0	10.0	15.0	100.0	75.0	200.0

**Table 2:** Effect of glass fiber powder concentration on the chromatic value of waterborne coatings with thermochromic ink from 18 °C to 40 °C.

Concentration of Glass Fiber Powder (%)	Chromatic Value	18 °C	20 °C	22 °C	24 °C	26° C	28 °C	30 °C	32 °C	34 °C	36 °C	38 °C	40 °C
Pure coating	*L*	84.0	84.8	85.3	85.3	84.3	84.7	83.8	83.8	83.8	83.9	85.6	84.6
	*a*	5.9	5.3	5.0	4.8	4.8	5.5	5.2	4.6	5.2	5.5	4.9	4.9
	*b*	28.5	28.7	29.6	29.5	27.8	28.4	28.1	27.0	27.7	27.2	28.5	28.3
	*C*	29.1	28.2	30.1	29.9	28.2	28.9	28.6	27.4	28.2	27.8	28.9	28.7
	*h*	78.2	79.3	80.3	80.6	80.1	79.0	79.3	80.3	79.2	78.5	80.1	80.1
0	*L*	65.8	64.3	66.6	66.7	66.2	68.0	72.5	76.6	79.4	79.9	81.2	81.6
	*a*	40.3	38.5	39.4	37.9	38.9	36.5	29.3	21.8	16.9	15.9	14.3	13.2
	*b*	24.9	24.5	25.6	25.4	25.1	24.9	24.5	25.1	26.3	26.4	27.4	27.8
	*C*	47.4	45.7	47.0	45.6	46.3	44.2	38.3	33.2	31.3	30.8	30.9	30.8
	*h*	31.7	32.4	33.0	33.8	32.8	34.3	39.9	49.0	57.2	58.8	62.4	64.5
1.0	*L*	65.5	65.5	65.8	65.1	65.8	66.1	67.4	74.1	77.3	78.1	78.4	79.1
	*a*	40.8	41.1	40.2	40.8	40.0	39.2	37.0	25.4	19.6	17.9	16.8	15.4
	*b*	26.0	25.6	25.4	25.6	25.3	25.3	24.4	23.5	24.5	24.8	24.6	25.6
	*C*	48.4	48.4	47.5	48.2	47.4	46.7	44.4	34.6	31.4	30.6	29.8	29.9
	*h*	32.5	31.9	32.3	32.1	32.3	32.8	33.4	42.7	51.3	54.2	55.6	58.9
3.0	*L*	68.5	68.6	68.7	69.4	69.2	68.7	69.5	73.9	79.0	79.5	80.8	81.1
	*a*	34.3	35.0	34.3	33.6	33.3	33.7	32.6	26.7	17.7	15.7	14.1	13.1
	*b*	27.5	27.7	27.9	28.1	28.2	27.6	28.1	26.7	27.9	28.8	29.0	29.6
	*C*	44.0	44.7	44.2	43.8	43.7	43.6	43.1	37.8	33.1	32.8	32.3	32.4
	*h*	38.7	38.4	39.1	39.9	40.2	39.3	40.7	45.0	57.5	61.3	63.9	66.0
5.0	*L*	65.5	65.6	65.7	65.6	66.0	65.9	68.4	74.7	77.0	77.9	78.6	79.0
	*a*	38.2	37.8	37.5	37.8	37.5	37.6	32.7	21.2	17.4	16.1	14.9	13.2
	*b*	24.0	23.8	24.1	23.8	23.9	24.1	23.4	23.6	24.3	24.3	24.8	25.2
	*C*	45.2	44.7	44.6	44.7	44.5	44.7	40.2	31.8	29.9	29.1	28.2	28.4
	*h*	32.1	32.1	32.7	32.1	32.5	32.6	35.5	48.0	54.3	56.3	59.4	62.3
7.0	*L*	67.4	67.4	67.3	67.5	67.6	68.1	68.6	73.5	77.3	77.8	78.8	79.1
	*a*	34.5	34.0	33.7	33.9	33.5	33.2	31.8	23.2	16.2	14.9	13.1	12.4
	*b*	25.0	25.4	25.3	25.2	25.5	25.0	24.5	24.0	25.3	25.2	26.1	26.1
	*C*	42.6	42.4	42.1	42.3	42.1	41.6	40.2	33.4	30.0	29.3	29.3	28.9
	*h*	35.9	36.7	36.9	36.6	37.3	36.9	37.6	46.0	57.4	59.3	63.3	64.4
10.0	*L*	67.6	67.5	67.1	67.5	67.0	67.5	68.0	68.9	73.3	74.9	75.6	76.3
	*a*	29.9	30.4	30.8	29.8	31.3	29.9	29.1	27.5	18.9	15.7	14.1	13.2
	*b*	24.4	24.2	24.1	24.4	23.8	23.9	23.7	22.3	23.8	24.0	24.6	24.9
	*C*	38.7	38.8	39.1	38.5	39.4	38.3	37.6	35.4	30.4	28.7	28.4	28.2
	*h*	39.2	38.5	38.0	39.3	37.2	38.6	39.1	39.0	51.5	56.8	60.1	62.1

**Table 3:** Effect of glass fiber powder concentration on the gloss of waterborne coatings with thermochromic ink.

Concentration of Glass Fiber Powder (%)	20° Gloss (%)	60° Gloss (%)	85° Gloss (%)
Pure coating	14.1	43.1	54.7
0	26.0	55.6	79.1
1.0	19.0	44.1	56.5
3.0	16.8	37.6	41.0
5.0	12.4	35.1	39.4
7.0	9.3	29.8	35.2
10.0	6.3	21.5	26.0

**Table 4:** Effect of glass fiber powder concentration on the adhesion of waterborne coatings with thermochromic ink.

Concentration of Glass Fiber Powder (%)	Damage Area (%)	Adhesion Level (level)
Pure coating	0	0
0	0	0
1.0	0	0
3.0	0	0
5.0	0	0
7.0	0	0
10.0	0	0

**Table 5:** Effect of glass fiber powder concentration on the impact resistance and hardness of waterborne coatings with thermochromic ink.

Concentration of Glass Fiber Powder (%)	Impact Resistance (kg·cm)	Hardness (H)
Pure coating	4.0	HB
0	5.0	H
1.0	5.0	H
3.0	5.0	2H
5.0	5.0	2H
7.0	5.0	2H
10.0	5.0	3H

**Table 6:** The chromatic value of waterborne coatings after liquid resistance test.

Concentration of Glass Fiber Powder (%)	Chromatic Value	Original	NaCl	Detergent	Ethanol	Red Ink
Pure coating	*L*	84.0	84.5	84.7	84.3	85.6
	*a*	5.9	6.1	6.2	6.6	6.9
	*b*	28.5	29.1	28.7	27.9	28.6
	*C*	29.1	30.2	30.8	27.8	29.9
	*h*	78.2	77.7	78.2	76.0	76.6
0	*L*	65.8	66.5	65.9	65.9	54.8
	*a*	40.3	39.3	39.0	39.5	70.2
	*b*	24.9	24.9	24.9	24.6	21.6
	*C*	47.4	45.7	45.5	42.8	73.5
	*h*	31.7	33.0	33.2	35.8	17.1
1.0	*L*	65.5	65.1	66.0	66.3	42.4
	*a*	40.8	40.1	39.9	40.1	73.0
	*b*	26.0	26.7	26.0	26.7	37.4
	*c*	48.4	48.2	47.7	48.3	82.0
	*H*	32.5	33.6	33.6	33.1	27.1
3.0	*L*	68.5	69.3	68.1	68.7	47.0
	*a*	34.3	33.8	35.1	33.9	74.6
	*b*	27.5	27.0	27.2	26.8	37.5
	*C*	44.0	43.2	44.8	43.3	83.5
	*h*	38.7	38.0	37.4	39.1	26.7
5.0	*L*	65.5	66.1	65.3	66.2	44.8
	*a*	38.2	37.6	38.6	37.8	73.8
	*b*	24.0	24.4	23.5	24.5	37.4
	*C*	45.2	44.0	45.9	44.7	82.8
	*h*	32.1	32.6	31.4	32.6	26.9
7.0	*L*	67.4	68.1	68.1	67.8	45.0
	*a*	34.5	34.1	33.7	33.8	72.4
	*b*	25.0	25.6	25.0	25.8	35.6
	*C*	42.6	41.2	41.6	41.1	80.7
	*h*	35.9	36.7	36.0	36.9	26.2
10.0	*L*	67.6	67.6	67.9	66.9	47.6
	*a*	29.9	30.4	29.4	30.3	73.0
	*b*	24.4	25.2	23.8	24.8	33.7
	*C*	38.7	39.5	38.4	39.2	80.4
	*h*	39.2	39.2	38.2	39.3	24.8

**Table 7:** Effect of glass fiber powder concentration on the color difference of waterborne coatings with thermochromic ink for liquid resistance.

Concentration of Glass Fiber Powder (%)	NaCl	Detergent	Ethanol	Red Ink
Pure coating	0.8	0.8	1.0	1.9
0	1.2	1.3	0.9	32.0
1.0	1.1	1.0	1.3	41.2
3.0	1.1	0.9	0.8	46.8
5.0	0.9	0.7	0.9	43.3
7.0	1.0	1.1	1.1	45.3
10.0	0.9	0.8	0.9	48.4

**Table 8:** Effect of glass fiber powder concentration on the level of waterborne coatings with thermochromic ink for liquid resistance.

Concentration of Glass Fiber Powder (%)	NaCl (Level)	Detergent (Level)	Ethanol (Level)	Red Ink (Level)
Pure coating	1	1	1	1
0	1	1	1	3
1.0	1	1	1	3
3.0	1	1	1	3
5.0	1	1	1	3
7.0	1	1	1	3
10.0	1	1	1	3

**Table 9:** Effect of time on the color values of heat preservation waterborne coatings.

Concentration of Glass Fiber Powder (%)	Color Value	18 °C	Three Months at 18 °C	30 °C	30 °C Heating 24 h	30 °C Heating 48 h	30 °C Heating 72 h
3.0	*L*	68.5	69.5	69.5	69.0	68.4	70.3
	*a*	34.3	33.6	32.6	33.2	33.5	31.5
	*b*	27.5	28.0	28.1	29.2	27.6	27.2
	*C*	44.0	43.8	43.1	42.7	42.9	42.3
	*h*	38.7	39.8	40.7	38.9	39.7	41.5

**Table 10:** Effect of time on the color difference of waterborne coatings.

Concentration of Glass Fiber Powder (%)	Color Difference after Three Months at 18 °C	Color Difference 30 °C Heating 24 h	Color Difference 30 °C Heating 48 h	Color Difference 30 °C Heating 72 h
3.0	1.4	1.3	1.5	1.6

**Table 11:** Gloss and chromatic values of waterborne wood coating before and after aging.

Sample	*L*	*a*	*b*	*C*	*h*	Δ*E*	Gloss (%)
Before aging	72.0 ± 0	19.3 ± 0	35.3 ± 0	40.3 ± 0	61.2 ± 0	–	35.9 ± 0
After aging	71.4 ± 0	22.1 ± 0	33.4 ± 0	40.2 ± 0	58.1 ± 0	3.4 ± 0	34.8 ± 0
